# Cutaneous Necrotizing Vasculitis and Leukopenia in a Cocaine User: Is Levamisole the Culprit?

**DOI:** 10.1155/2016/2685267

**Published:** 2016-08-07

**Authors:** Lara El Khoury, Nabil Zeineddine, Richard Felix, Mark Goldstein

**Affiliations:** ^1^Internal Medicine Department, Staten Island University Hospital, 475 Seaview Avenue, Staten Island, New York, NY 10305, USA; ^2^Pathology Department, Staten Island University Hospital, 475 Seaview Avenue, Staten Island, New York, NY 10305, USA; ^3^Rheumatology Department, Staten Island University Hospital, 475 Seaview Avenue, Staten Island, New York, NY 10305, USA

## Abstract

Levamisole is an antihelminthic drug banned by the US Food and Drug Administration (FDA) in 2000 because of its dangerous side effects. Over the past few years, it has been identified as an adulterant in cocaine and reported to cause cutaneous vasculitis in cocaine users. The health burden of levamisole is serious since it is estimated that over 5 million Americans use cocaine and that 70% of the cocaine used in the USA contains levamisole. In this paper we report the case of a 23-year-old female cocaine user that presented with purpuric rash and skin necrosis, found to have positive c-ANCA and anti-proteinase 3 antibodies. Her skin biopsy showed fibroconnective tissue with signs of necrosis, acute and chronic inflammation, and thrombus formation. She was diagnosed with levamisole-induced vasculitis and successfully treated with withdrawal of cocaine use and local wound care.

## 1. Introduction

Levamisole is a veterinary antihelminthic drug that was initially approved for the treatment of several conditions including certain malignancies and rheumatoid arthritis [1, 2].

In 2000, the US Food and Drug Administration (FDA) banned the use of levamisole in the United States (US) because of dangerous side effects such as agranulocytosis and skin necrosis [3, 4].

Over the past few years, levamisole has been identified as an adulterant in cocaine, used to add weight and volume to the drug. Since 2010, numerous reports have emerged describing cutaneous vasculitis in cocaine users.

We describe a case of a 25-year-old female patient, cocaine user, presenting with painful dark discoloration of lower extremities.

## 2. Case Presentation

A 23-year-old African-American female patient presented to the hospital for painful skin lesions over her lower extremities and neck.

Her past medical history includes cocaine and heroin abuse and hepatitis C virus infection for which she never received any treatment. She smokes one pack of cigarettes and consumes 2-3 alcoholic drinks per day. Patient snorts cocaine on a daily basis, and her last use of cocaine occurred one day prior to her presentation.

The onset of the skin lesions was acute, started seven days prior to presentation, when she started complaining of burning sensation over the anterior shins and retroauricular areas.

**Figure 1:**
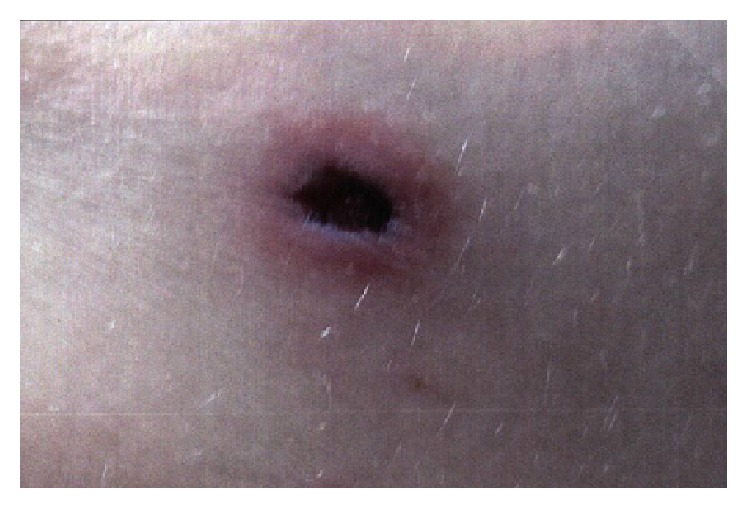
Lesion over the neck.

**Figure 2:**
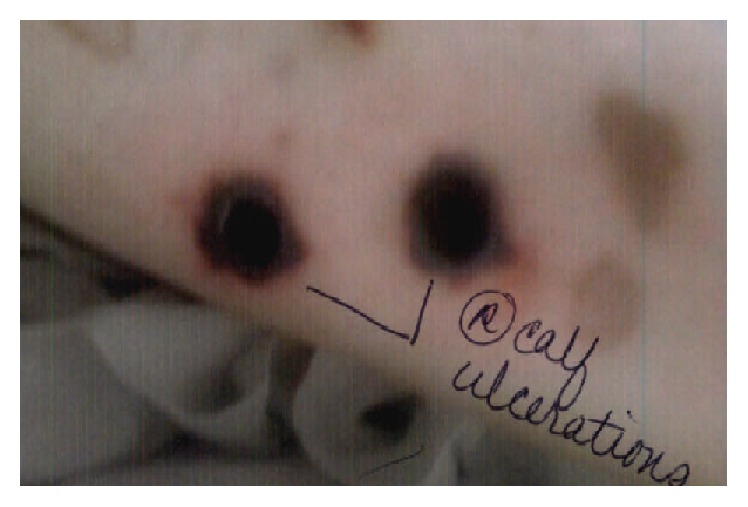
Right calf lesions.

**Figure 3:**
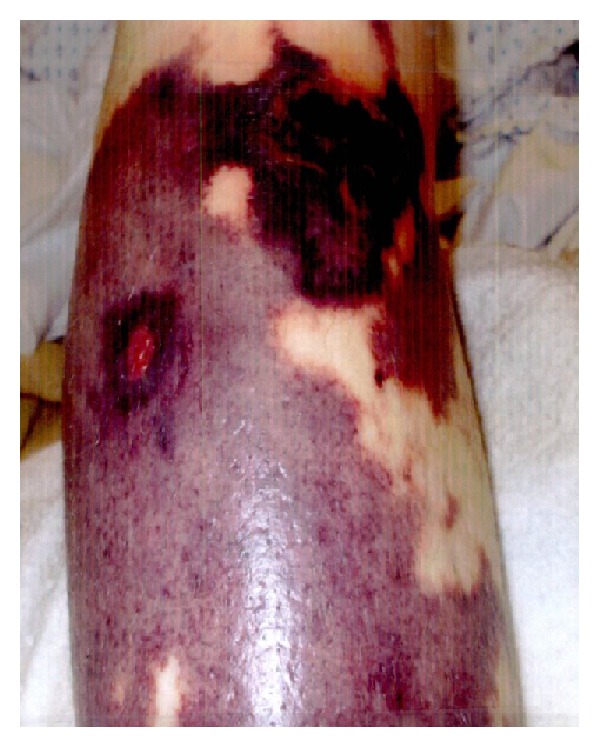
Extensive involvement of the leg.

On presentation, patient had a temperature of 99.7 F, blood pressure of 118/67 mmHg, heart rate of 90/min, and respiration rate of 16/min. On physical exam, she had marks of prior needle injections over bilateral arms and forearms. She had hyperpigmented skin lesions over the nape of the neck, retroauricular areas, with palpable collection beneath the skin at the posterior aspect of the neck. Examination also revealed the presence of numerous, bilateral, anterior, and posterior lower extremities skin lesions, of varying sizes, purplish in color with erythematous border, nonblanching, tender, with necrotic center (Figures 1, 2, and 3). Oral mucous membranes were normal, and the rest of the physical exam was unremarkable.

The review of system was negative for headache, oral ulcers, fever, chills, hemoptysis, cough, or hematuria.

Upon admission, laboratory findings included a white blood cells count of 2300, hemoglobin level of 12.2 mg/dL, and platelets of 228000, and her basic metabolic profile and liver function tests were normal. Her urine drug screen was positive for cocaine and opiate.

Based on this presentation and her history of cocaine use and hepatitis C, concern was for systemic vasculitis versus levamisole related vasculitis. Erythrocyte sedimentation rate (ESR) was elevated at 66 mm/hr; her HIV serology was negative as were her serum cryoglobulins level, lupus anticoagulant, B2 glycoprotein, and anticardiolipin antibodies. Her p-ANCA and anti-myeloperoxidase antibodies were negative, but her c-ANCA and anti-proteinase 3 antibodies were positive at a level of 3.8 AI (reference range < 1 AI).

**Figure 4:**
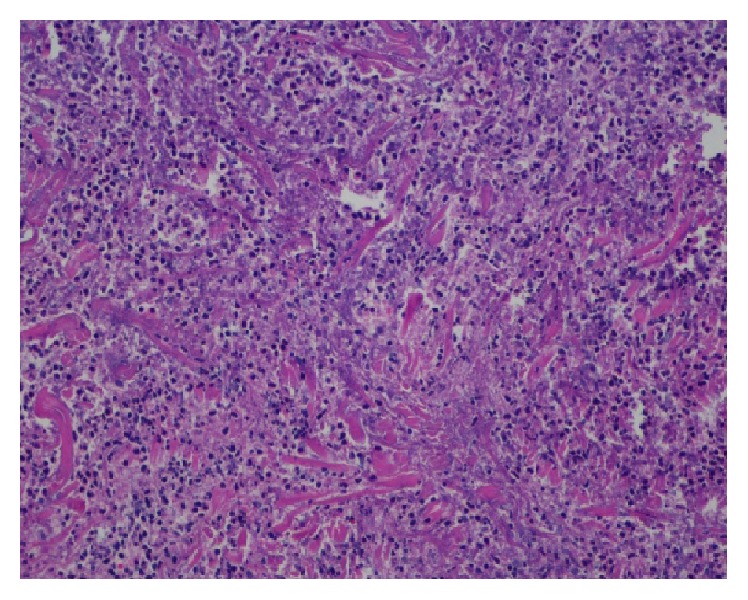
Perivascular inflammation.

**Figure 5:**
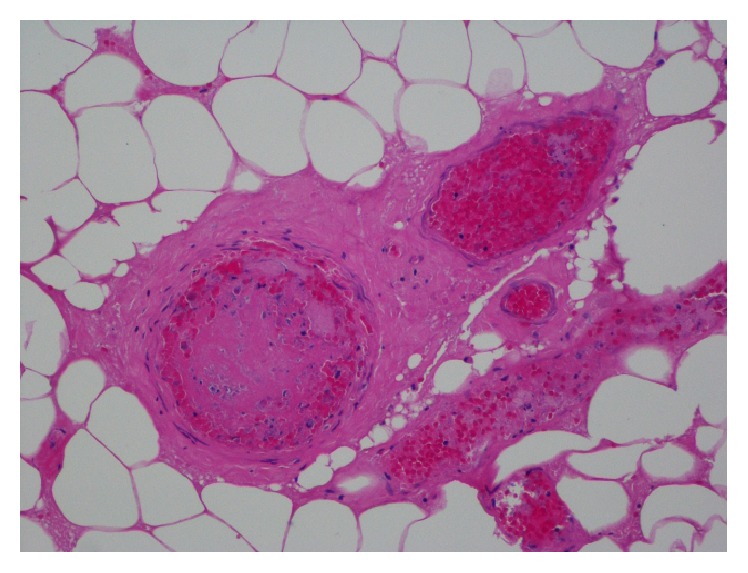
Fibrin thrombus formation.

A biopsy of one of her shin skin lesions was performed and revealed the presence of fibroconnective tissue with signs of necrosis, acute and chronic inflammation, and thrombus formation (Figures 4 and 5).

Based on the clinical presentation as well as the laboratory and pathology findings, she was diagnosed with levamisole-induced vasculitis complicated by abscess formation at the level of the neck, associated with levamisole-induced bone marrow suppression and leukopenia.

She was counseled at length about cessation of cocaine use as it is the major beneficial therapeutic measure for her condition. She was not given corticosteroids. Her neck abscess was drained and she received a course of antibiotherapy. She attended individual and group counseling during her admission. The patient was prescribed needed medications to prevent any withdrawal symptoms and discharged with the instructions to follow up with the detoxification clinic and with a rheumatologist. Upon follow-up evaluation, her skin lesions significantly improved with interruption of cocaine use.

## 3. Discussion

According to a national survey, more than five million Americans abuse cocaine. Around 70% of the cocaine used in the US contains levamisole which is identified as a common adulterant believed to add weight and volume to the drug and possibly potentiate its effects [5, 6]. Since 2010, numerous reports have emerged describing a characteristic vasculitis in patients who abuse cocaine [4, 7–10].

Most cases of levamisole-induced vasculitis were described in middle-aged (mean age 44 y) women (male : female ratio of 1 : 3) [11].

The typical presentation includes the development of multiple purpuric, reticulated, tender skin lesions over the nose, cheeks, ears, trunk, and extremities [4, 12]. These skin lesions can develop central necrosis, hemorrhagic bulla complicated by subsequent skin sloughing.

Our patient had developed the characteristic tender, purpuric, necrotic skin lesions over her bilateral extremities as well as over the neck and behind the ears. The neck lesions were complicated by an abscess formation. In fact, it is not uncommon for the skin lesions in vasculitis to develop bacterial superinfection.

Along with the skin manifestations, levamisole-induced vasculitis has been associated with certain laboratory findings such as leukopenia, neutropenia, agranulocytosis, elevated ESR, and positive antibodies such as p- and c-ANCAs; antinuclear antibody and lupus anticoagulant [4, 13, 14]. In our case, the patient had leukopenia, elevated ESR, and positive c-ANCA antibodies.

In terms of the pathologic findings, two classic features are commonly recognized: a leukocytoclastic vasculitis affecting the small vessels with fibrinoid necrosis and a thrombotic pattern with fibrin thrombi within the vessels. The latter being the most consistent pattern [11]. The skin biopsy from the neck lesions of our patient revealed multiple fibrin deposition within the small vessels with signs of necrosis.

It is important to keep in mind that all ANCA-associated vasculitides (granulomatous polyangiitis, microscopic polyangiitis, and Churg Strauss) present with palpable purpuric skin rash similar to levamisole-induced vasculitis. Moreover, the pathologic findings of leukocytoclastic vasculitis and fibrin thrombi can be similar. This makes the clinical correlation crucial for making the diagnosis of levamisole-induced vasculitis.

The treatment options for this condition remain limited. In fact, it has been well established that the majority of the patients have significant resolution of their skin lesions after cessation of cocaine use [11, 13]. On the other hand, corticosteroids have been used for resistant cases with conflicting results [15]. A major complication often seen in these patients is the development of bacterial superinfection of the skin lesions that might require in some cases surgical debridement and possible skin grafting and extensive wound care.

Our patient's neck lesions were complicated by abscess formation that required drainage and antibiotherapy. Fortunately, she had a significant improvement and healing of the skin lesions after interruption of cocaine abuse.

## 4. Conclusion 

Levamisole-induced vasculitis is a recently established entity associated with the use of levamisole adulterated cocaine. The clinical picture includes a cutaneous vasculitis syndrome characterized by the development of multiple purpuric often necrotic skin lesions over the extremities and face. The majority of the patients develop neutropenia or agranulocytosis, along with an elevated ESR and positive autoantibodies such as c- or p-ANCAs, ANA, and lupus anticoagulant.

This constellation of findings coupled with the pathologic features of leukocytoclastic vasculitis and/or microvascular thrombi can mimic the presentation of the ANCA associated vasculitides. Therefore, the clinician should have a high index of suspicion for this entity in the setting of a history of cocaine abuse especially that resolution of the symptoms can be achieved with cessation of cocaine abuse solely.
